# Performance Insights in Speed Climbing: Quantitative and Qualitative Analysis of Key Movement Metrics

**DOI:** 10.3390/bioengineering12090957

**Published:** 2025-09-06

**Authors:** Dominik Pandurević, Paweł Draga, Alexander Sutor, Klaus Hochradel

**Affiliations:** Institute of Measurement and Sensor Technology, UMIT TIROL-Private University for Health Sciences and Health Technology, 6060 Hall in Tirol, Austria; pawel.draga@umit-tirol.at (P.D.); alexander.sutor@umit-tirol.at (A.S.); klaus.hochradel@umit-tirol.at (K.H.)

**Keywords:** computer vision, machine learning, deep learning, convolutional neural networks, sports biomechanics, sports science in sports, kinematic and performance analysis, sport biomechanics, training optimization, Speed Climbing

## Abstract

This study presents a comprehensive analysis of Speed Climbing athletes by examining motion parameters critical to elite performance. As such, several key values are extracted from about 900 competition recordings in order to generate a dataset for the identification of patterns in athletes’ technique and efficiency. A CNN-based framework is used to automate the detection of human keypoints and features, enabling a large-scale evaluation of climbing dynamics. The results revealed significant variations in performance for single sections of the wall, particularly in relation to start reaction times (with differences of up to 0.27 s) and increased split times the closer the athletes are to the end of the Speed Climbing wall (from 0.39 s to 0.45 s). In addition, a more detailed examination of the movement sequences was carried out by analyzing the velocity trajectories of hands and feet. The results showed that coordinated and harmonic movements, especially of the lower limbs, correlate strongly with the performance outcome. To ensure an individualized view of the data points, a comparison was made between multiple athletes, revealing insights into the influence of individual biomechanics on the efficiency of movements. The findings provide both trainers and athletes with interesting insights in relation to tailoring training methods by including split time benchmarks and limb coordination.

## 1. Introduction

In the past two decades, climbing has transformed from a niche activity to a rapidly expanding global sport. The International Federation of Sport Climbing (IFSC) estimates that approximately 25 million people worldwide participate regularly in climbing, reflecting its power of attraction across different age groups and regions. In the time between 2001 and 2012, the global number of climbing facilities and climbers increased by almost 50% [1]. The sport’s ascent is further accelerated by its growing media presence and its recognition on the international stage, including its debut at the 2020 Olympic Games in Tokyo [2]. This event showcased the sport’s unique combination of athleticism, strategy, and creativity, captivating audiences and inspiring a new generation of climbers. Its continued inclusion in Paris 2024 has solidified climbing’s status as a mainstream sport, with the potential to attract even more participants and fans. Since its inclusion in the Olympic program, the competition format and scoring system in sport climbing have undergone significant transformations, resulting in Speed Climbing being established as a separate discipline [3].

Looking ahead, the Olympic Games 2028 in Los Angeles will mark a significant milestone for the sport; for the first time, all three disciplines will be held as fully separated events, each awarding its own set of medals. This new format will allow the competing countries to win up to three medals in climbing, further enhancing the sport’s visibility and providing greater recognition for the unique skills required in each discipline.

These changes reflect the specific demands of each climbing discipline. Recent studies highlight substantial differences in exercise characteristics and physiological load [4,5], emphasizing the unique requirements of Speed Climbing. Draga et al. [6] highlight the importance of optimizing body composition and proportions for peak performance in this discipline. Krawczyk et al. [7] underline the crucial role of biomechanical parameters such as limb strength and power.

Particularly in Speed Climbing, performance has seen remarkable advances. At the Paris 2024 Olympics, both men’s and women’s records were shattered, with finishing times dropping from 6.97 s to 6.06 s for women and from 5.45 s to 4.74 s for men [8], signaling a breakthrough after stagnation in the years following Tokyo 2020.

These improvements are attributed to movement efficiency, such as the optimization of motion patterns in the start section and route strategies, as well as enhanced training methods [9]. To maintain this rapid progress and further enhance the performance of Speed Climbing athletes, the usage of measurement technology and automated analysis tools is essential. These systems provide precise, objective data that support both researchers investigating biomechanics and exercise physiology, as well as practitioners, who make informed decisions and optimize training methods [10]. Tools such as video analysis and motion tracking systems have already been successfully integrated in well-established sports, offering valuable insights into athletic performance, identifying areas for improvement, and enabling coaches to tailor training programs more effectively. Their demonstrated success in other sports [11,12] highlights the potential for accelerating the development of sport climbing.

Recent advancements in sports science have been driven by the development of automated video analysis systems through the integration of machine learning and computer vision techniques. This has enabled a better understanding of athletic performance across different sport disciplines and helped to prevent injuries by identifying risky movement patterns early on [13]. In particular, the use of Convolutional Neural Networks (CNNs) for human pose estimation has revolutionized the field and has become a cornerstone for biomechanical analysis, allowing for the extraction of various kinematic and kinetic parameters from video recordings [14]. Unlike sensor-based methods, which require athletes to wear additional equipment and can affect natural movement, CNN-based approaches utilize only video data, providing a completely non-invasive and practical solution with no extra effort needed from athletes or trainers. The application of deep learning and CNNs is distinguished, among other characteristics, by its ability to automatically extract features from video data, outperforming traditional handcrafted feature approaches in a variety of sports contexts. In soccer, for instance, CNN-based frameworks have been successfully deployed for event detection and classification, enabling the identification of tactical patterns and key actions in video sequences [15]. In a more general context, the deployment of CNNs has shown significant improvements in the analysis of human motion. In upper limb motion analysis, CNNs have demonstrated high accuracy in classifying movement types and detecting kinematic differences between healthy study participants and participants who have had a stroke, providing objective and reproducible assessments that surpass conventional observation methods [16]. Moreover, CNN-based models are increasingly being used to analyze gait and limb coordination, amongst others, in athletic settings [17]. The integration of such technologies in sports not only enhances performance analysis but also opens new avenues for personalized feedback and targeted training deployments. Building on methodological advances in movement analysis and the successful application of CNNs across various sports, this study aims to leverage these technologies to gain deeper insights into coordination and performance in Speed Climbing.

Since the introduction of Speed Climbing as an Olympic discipline, the interest in performance analysis and optimization has grown significantly and so has the research in this area. For example, one study has demonstrated how video analysis can provide detailed insights into trajectory efficiency, coordination strategies, and movement fluency among elite climbers, offering non-invasive means to access different aspects of performance [18]. While traditional video analysis methods have provided valuable insights into movement strategies and efficiency, advances in deep learning have enabled even more precise results. A framework based on 3D Residual Networks that was proposed in [19] presents the automated classification and analysis of Speed Climbing videos. Using an extensive annotated dataset of recorded runs, this approach applies 3D convolutions and residual connections to capture and evaluate different climbing state combinations. Extending these foundations, the following presented approach uses CNN-derived motion parameters to provide a more granular understanding of Speed Climbing performance.

This paper presents an analysis of movement patterns in Speed Climbing using a comprehensive dataset consisting of nearly 900 competition recordings involving approximately 250 athletes. Both quantitative and qualitative evaluations are conducted to gain deeper insights into the performance of Speed Climbing athletes. The study investigates the hypothesis that the athletes’ split times and limb coordination are decisive factors for success and, therefore, correlate with the end time. To be precise, the following research questions are addressed:

- How can the analysis of split times and comparison with elite speed climbers serve as a basis for targeted adjustments to training methods that lead to measurable improvements in performance?
- Does a proper coordination of individual limb movements correlate with competitive success in Speed Climbing?

Based on this, the methods used to determine the mentioned movement parameters are first presented, followed by a detailed analysis of the results. The findings are then discussed in the context of the existing literature, as well as their implications for training and performance optimization in Speed Climbing being highlighted.

## 2. Materials and Methods

The analysis of climbing movements is performed using a CNN-based framework for the automated detection of human keypoints and features on single frames [9]. The framework is trained on a dataset of annotated Speed Climbing videos, allowing it to accurately identify and track the positions of key body joints during climbing movements, as well as different features, on every frame. This enabled the extraction of relevant motion parameters, such as limb angles, joint velocities, or contact times. The framework is applied to a collection of about 900 competition recordings, including a diverse range of athletes and climbing styles. The resulting dataset, consisting of several motion-describing parameters, is carefully filtered and sorted, ensuring a representative wide variety and allowing for a comprehensive analysis of the athletes’ performance.

### 2.1. Data Acquisition and Postprocessing

The data acquisition process involved the collection of video recordings from various Speed Climbing competitions over a period from 2017 to 2024. The Speed Climbing runs from qualification to finals of 29 IFSC competitions are included and analyzed on authorized platforms, in accordance with an IFSC approval process granted specifically for research purposes.

The videos are processed using the CNN-based framework, combining MMPose/MMDet [20,21] for the detection of human keypoints and Yolov8 [22] for the recognition of Speed Climbing holds on every frame. As such, pretrained models are fine-tuned on custom annotated datasets from 17 different Speed Climbing videos, ensuring domain-specific accuracy. In evaluating the detection performance of the frameworks, the Mean Average Precision (mAP) metric is used, yielding values of >95% for both human body (MMDet) and hold detection (Yolov8), indicating high reliability in the context of Speed Climbing movement analysis.

In addition to these models, an Optical Flow algorithm was applied to determine the movement of the camera during the recording and, consequently, the absolute position of the athletes on the wall.

**Figure 1:**
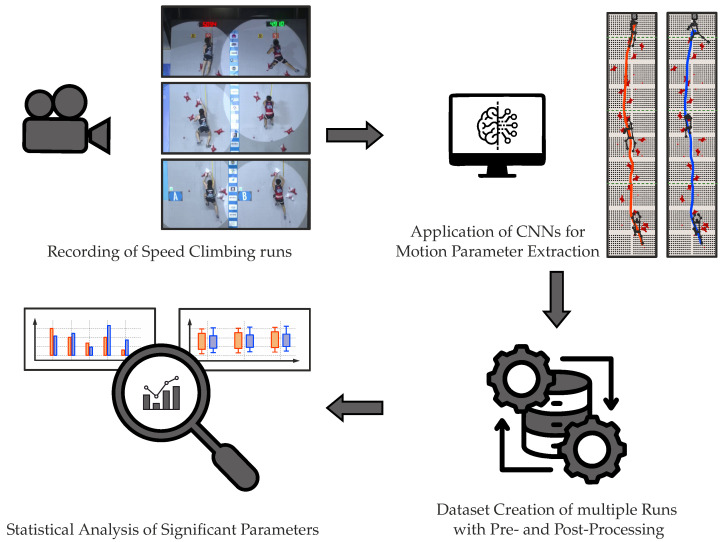
Visualization of the methodology from Speed Climbing recordings, through the CNN-based extraction of motion parameters from multiple runs, to dataset creation and the statistical analysis of significant performance parameters.

Following the flow chart describing the methodology of this study in Figure 1, a dataset is created consisting of 861 videos with 1717 runs and 248 different athletes (120 female and 128 male). The video resolutions of the analyzed Speed Climbing runs vary between 720 p and 1080 p, with a frame rate of 25 up to 30 Hz. Additionally to the extracted motion-describing parameters like COG dynamics or limb frequencies, the following information is manually recorded for each video/athlete:

- Competition name;
- Competition date;
- Qualification/Finals round;
- Athlete name;
- Technique used in the start section;
- Achieved end time.

In a final step, the results of each processed video or run are evaluated in order to obtain the dataset for further analysis. Therefore, the end time $t_{m}$ calculated using the automated framework is compared with the actual documented finishing time $t_{gt}$ of the athlete. To estimate the characteristic time $t_{m}$ marking the buzzer touch during the final jump, the velocity profile $v_{c}\left(t\right)$ of the athlete’s center of gravity (COG) is analyzed within a defined region of interest (ROI). This ROI spans from $t_{1}$ (when the COG’s vertical position intersects the last hand hold) to $t_{2}$ (initiation of downward body movement after touching the buzzer). A manual validation of several recordings and a comparison of the CNN framework results revealed that $t_{m}$ corresponds to the inflection point of the last decay phase of the COG velocity, where the second derivative of velocity crosses zero, as follows:

(1) $$\begin{array}{c} {\left.\frac{d^{2}v_{c}}{dt^{2}}\left(t\right)\right|}_{t=t_{m}}=0\;\mathrm{for}\;t_{1}<t_{m}<t_{2}. \end{array}$$

To ensure the accuracy of the calculated results and thus the reliability of the dataset, a deviation of 5% from the ground truth end time $t_{gt}$ is set, as follows:

(2) $$\begin{array}{c} \left|t_{m}-t_{gt}\right|\leq 0.05\cdot t_{gt}. \end{array}$$

The 5% error threshold is empirically determined by considering several possible sources of uncertainty. These include the manual identification of the start frame and potential inaccuracies in detecting the exact moment of buzzer contact. Additionally, single-frame errors can arise from the recording camera setups, where slight distortions could occur in the final meters of the Speed Climbing wall due to missing intrinsic calibration parameters.

Dataset rows failing to meet this criterion are removed, resulting in a filtered dataset of 1338 valid Speed Climbing runs used for further analysis.

### 2.2. Data Analysis

The preprocessed and filtered dataset is analyzed using several statistical methods to identify patterns and correlations between the athletes’ performance and the extracted motion parameters. The analysis focused on the following key aspects: a quantitative evaluation of the athletes’ performance, including the distribution of split times, and limb velocity trajectories. For both these parameters, qualitative comparisons are made including, on one hand, morphologically distinct athletes to highlight the influence of individual biomechanics and, on the other hand, athletes with different performance outcomes (e.g., comparison with current world record holders).

Descriptive statistics are calculated for athletes using the so-called Tomoa Skip in the start section, achieving end times below 6 (male) and 7 (female) seconds. The dataset is thus further reduced in order to avoid scattering due to outliers arising from different techniques or performance groups. For a more detailed explanation, the calculation and the corresponding analysis of the mentioned parameters are carried out in the following sections.

#### 2.2.1. Split Times Analysis

The split times are calculated for each athlete and each run, allowing for an analysis of the performance in individual sections of the wall. The split times refer to the absolute time values an athlete takes to move their COG to the next hand hold, which is measured from the moment the athlete’s COG intersects the height of the hold. This results in a total of 20 split times per run, mapping the athlete’s movement trajectory and enabling a detailed analysis of pacing, efficiency, and technique.

To compare these split times effectively and identify general performance weaknesses/strengths, the relative time differences are adjusted by dividing them by the respective vertical distance traveled between two consecutive hand holds, as follows:

For i=1,2,…,20:

(3) $$\begin{array}{cc} \Delta t_{i} & =t_{i}-t_{i-1} \end{array}$$

(4) $$\begin{array}{cc} \Delta h_{i} & =h_{i}-h_{i-1} \end{array}$$

(5) $$\begin{array}{cc} \Delta {\hat{t}}_{i} & =\frac{\Delta t_{i}}{\Delta h_{i}} \end{array}$$

This allows the normalized relative time $\Delta {\hat{t}}_{i}$ to be calculated for Holds 1 to 20. Therefore, $t_{1,\ldots ,20}$ are the absolute time values of the athlete’s COG crossing the respective holds, while $h_{1,\ldots ,20}$ are the corresponding heights of the holds on the Speed Climbing wall. The value $t_{0}$ defines the athlete’s movement initiation, while $h_{0}$ denotes the vertical position of the athlete’s COG at this time. In general, this normalization allows for a more accurate assessment of the athletes’ performance, as it considers the varying distances between holds and provides a standardized measure.

Additionally, two non-normalized metrics are included due to their significant dependency on the athlete’s physics and used techniques in the start and end sections. These are as follows:

- Reaction Time $t_{r}$: Time between start signal ($t_{s}$, manually annotated) and movement initiation ($t_{0}$):
  (6) $$\begin{array}{cc} t_{r} & =t_{0}-t_{s} \end{array}$$
- Jump Time $t_{j}$: Time from the last hold ($t_{20}$) to triggering the end buzzer ($t_{gt}$):
  (7) $$\begin{array}{cc} t_{j} & =t_{gt}-t_{20} \end{array}$$

To establish a relationship between these calculated times and the athletes’ physical characteristics, selected anthropometric parameters are included. The measurements of, among others, height, arm span, weight, and body mass index (BMI) were recorded according to standardized protocols [23] using a *Siber-Hegner* anthropometer (Silber Hegner & Co. Ltd., Zurich, Switzerland) for the body height and arm span and a *Tanita TBF 583* scale (Tanita Corporation, Tokyo, Japan) for the body weight. The data were recorded during competitions at different times of the day, taking into account the pre-competition context. To assess repeatability and reliability, the technical error of measurement (TEM) was calculated based on three non-consecutive measurements; this remained below 1% for all parameters. The anthropometric assessments were performed by the same trained researcher and were conducted voluntarily and anonymously, ensuring the athletes’ privacy and data protection.

#### 2.2.2. Limb Coordination Analysis

For the investigation of limb coordination, the velocity trajectories of hands and feet were analyzed. These velocities are calculated based on the detected keypoints of the athletes’ limbs and the corresponding time intervals. Due to the highly dynamic nature of Speed Climbing and the synchronized limb movements—alternating between holds by accelerating and decelerating key body points—it is hypothesized that the velocity trajectories of hands and feet follow a sinusoidal pattern. The analysis of these velocity profiles provides valuable insights into the athletes’ movement patterns, coordination during climbing, and any possible correlations with their performance.

To verify this assumption, the calculated and extracted velocity data of hands and feet are fitted to a sinusoidal function using a least-squares fitting approach. As such, the velocity signal of the selected joint is extracted from the dataset and a relevant time window for the analysis is defined in order to ensure that irregularities at the start and end of the movement are excluded. Therefore, the first velocity peak of each respective limb was selected as start index, and the last peak was chosen as the end index. The velocity data v(t) are then fitted to a sinusoidal model of the following form:

(8) $$\begin{array}{c} \tilde{v}\left(t\right)=A\cdot \sin\left(\omega t+\varphi \right)+C, \end{array}$$

with *A* as amplitude, ω as angular frequency, ϕ as phase shift, and *C* as vertical offset. The fitting is performed using a least-squares approach, where the optimal parameters θ=[A,ω,φ,C] in $\tilde{v}\left(t\right)$ are predicted by minimizing the sum of squared residuals (SSR) [24] between the observed velocity data v(t) and the fitted model, as follows:

(9) $$\begin{array}{c} \min_{\theta }\;\mathrm{SSR}=\min_{\theta }\sum_{i=1}^{N}{\left[v\left(t_{i}\right)-\left(A\cdot \sin\left(\omega t+\varphi \right)+C\right)\right]}^{2}. \end{array}$$

To obtain the best optimization results, the choice of initial parameters is crucial. Since velocity data typically revolve around the mean value, the amplitude *A* and offset *C* are set. By selecting the time window described above, the initial value of the phase shift φ can be set to zero. For the frequency of the sinusoidal model, $f=\frac{\omega }{2\pi }$, two approaches are tested, as the choice of this initial value has a significant impact on the optimization results. On the one hand, the frequency of the velocity signal can be estimated by investigating the so-called instantaneous frequency. The instantaneous frequency approach estimates a signal’s frequency at defined points in time, providing a more detailed representation of the signal’s frequency content. This method is particularly useful for analyzing non-stationary signals, where the frequency content may change over time. The instantaneous frequency is calculated using the Hilbert transform, which provides an analytic signal representation. The instantaneous frequency f(t) is then given as follows [25]:

(10) $$\begin{array}{c} f\left(t\right)=\frac{1}{2\pi }\frac{d}{dt}\varphi \left(t\right). \end{array}$$

The resulting frequency response f(t) is averaged over the defined time window to obtain a single frequency value as an initial guess for the optimization problem. However, since the recordings, and thus the data, are limited to low sampling rates (25–30 Hz), the length of the frequency vector is significantly reduced, resulting in a high scatter of these values and a less reliable estimation using the mean.

The second approach is based on the assumption that the frequency of the velocity signal is approximately constant and can be defined through an investigation of the frequency spectrum of the signal. The frequency spectrum is obtained by applying a Fast Fourier Transform (FFT) to the velocity data, which decomposes the signal into its frequency components. The resulting frequency spectrum is then analyzed to identify the dominant frequency with the highest energy contribution. For further refining of the initial guess, this dominant frequency $f_{d}$ is used as a starting point for an iterative minimization of the SSR. Therefore, the best optimization result is determined within a defined range of frequencies (±20% around $f_{d}$).

For the validation of the fitting results, the goodness of fit is evaluated using the coefficient of determination $R^{2}$ [24], which quantifies the proportion of variance in the observed data that can be explained by the following fitted model:

(11) $$\begin{array}{c} R^{2}=1-\frac{\mathrm{SSR}}{\mathrm{SST}}, \end{array}$$

with SST being the sum of the squared total deviations, as follows:

(12) $$\begin{array}{c} \mathrm{SST}=\sum_{i=1}^{N}{\left[v\left(t_{i}\right)-\bar{v}\right]}^{2} \end{array}$$

and $\bar{v}$ being mean of the observed velocity data. The $R^{2}$ value indicates how well the fitted model explains the variability in the observed data, with values closer to 1 indicating a better fit.

The fitted sinusoidal model is then used to analyze the velocity trajectories of hands and feet during climbing movements, providing insights into the athletes’ coordination and movement patterns.

In addition to the analysis of individual limb kinematics, the quantification of inter-limb coordination is crucial for identifying synchronization patterns underpinning movement efficiency. To objectively assess anti-synchronicity (oppositional motion) or synchronicity (in-phase coordination), the instantaneous phase differences Δφ(t) between limb velocity signals are calculated using the Hilbert transform H. For each signal v(t), the analytic representation $z_{v}\left(t\right)$ and the corresponding phase distribution φ(t) can be computed as follows [25]:

(13) $$\begin{array}{cc} z_{v}\left(t\right) & =v(t)+i\cdot H(v(t)), \end{array}$$

(14) $$\begin{array}{cc} \varphi (t) & =\arg\left(z_{v}\left(t\right)\right). \end{array}$$

After calculating the absolute differences between the phase distributions of two different limbs $\Delta \varphi \left(t\right)=\left|\varphi_{1}\left(t\right)-\varphi_{2}\left(t\right)\right|$ and wrapping the angles to the interval [0,2π], the quantification of inter-limb coordination is performed by calculating the mean absolute deviation from π, as follows:

(15) $$\begin{array}{c} \mu_{\pi }=\frac{1}{N}\sum_{i=1}^{N}\left|\Delta \varphi (t_{i})-\pi \right|. \end{array}$$

Therefore, low values of $\mu_{\pi }$ indicate anti-synchronicity, while higher values suggest synchronicity. This measure provides a quantitative assessment of the coordination between limbs during climbing movements. By linking phase dynamics to performance outcomes, this approach reveals how optimal inter-limb timing enhances athletic execution and efficiency.

## 3. Results

Using the dataset generated and filtered as described above, consisting of 1338 valid Speed Climbing runs, an evaluation of the defined parameters is performed and the results are presented in the following sections. Therefore, the focus is set on the analysis of the part of the dataset including male athletes, using the Tomoa Skip technique in the start section and achieving end times below 6 s. This results in a total of 321 runs with 63 different athletes. By limiting the analysis to this specific group of male elite athletes, the influence of different techniques and performance levels is minimized, allowing for a more accurate assessment of the athletes’ movement patterns and performance characteristics. The results are presented in two sections. First, the quantitative analysis of the split times is presented, followed by a qualitative analysis of the limb coordination and velocity trajectories.

### 3.1. Split Times Analysis

**Figure 2:**
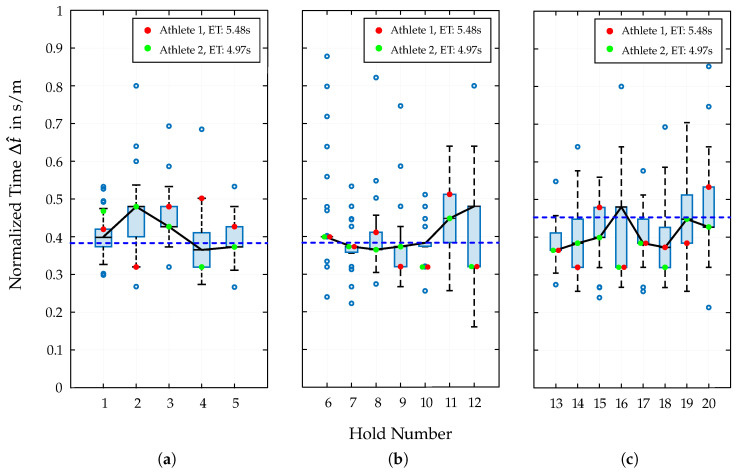
Distribution of the normalized relative split times $\Delta \hat{t}$ to each hold (1–20) of the Speed Climbing wall divided into the start (**a**), middle (**b**), and end (**c**) section. The red and green dots indicate the corresponding split times of two athletes with different morphological characteristics and performance outcomes. The dashed blue line indicates the average value, while the black straight line depicts the course of the median for $\Delta \hat{t}$ per section.

For the evaluation of the split times in Speed Climbing, the described dataset is used to perform a quantitative analysis of the individual wall sections. The results are presented in Figure 2, which shows the distribution of the normalized relative split times $\Delta \hat{t}$ to each hold (1–20) of the Speed Climbing wall, as calculated using Equation (5).

Additionally, data points from two athletes are included, and their distribution within the overall dataset is examined. These two athletes differ significantly in their physical characteristics, especially by height and arm span, as well as in their performance outcome (see Table 1). For each athlete, the best run from the analyzed dataset was used.

An interesting observation, although expected, is the standard deviation of split times in the start section (σ≈0.028s/m) compared to the middle (σ≈0.033s/m) and end sections (σ≈0.035s/m) of the Speed Climbing wall. By using the same movement pattern (Tomoa Skip), the athletes only differ in the execution of the motion sequences. In the area from Hold 3 to Hold 4, the most noticeable differences can be identified at the moment of re-accelerating from the so-called deep squat position. Therefore, the athlete experiences a drop in speed through low COG positions as a result of the path shortening in this section.

Also noticeable is the highly non-uniform distribution during the transfer from the start to middle section and, thus, the arrangement of split times towards Hold 6. While more than 60% of the data points are concentrated at $\Delta \hat{t}\approx 0.4\;s/m$, a large number of outliers are distributed around this value, with a wide dispersion. Significant variations in split times and movement execution are also observed in the region from Hold 9 to 12. Therefore, performing a jump from Hold 9 to 11 results in scattered data points, which are mainly influenced by the morphological characteristics of the athletes, which affect both foot positioning on the wall and the trajectory of the COG.

**Figure 3:**
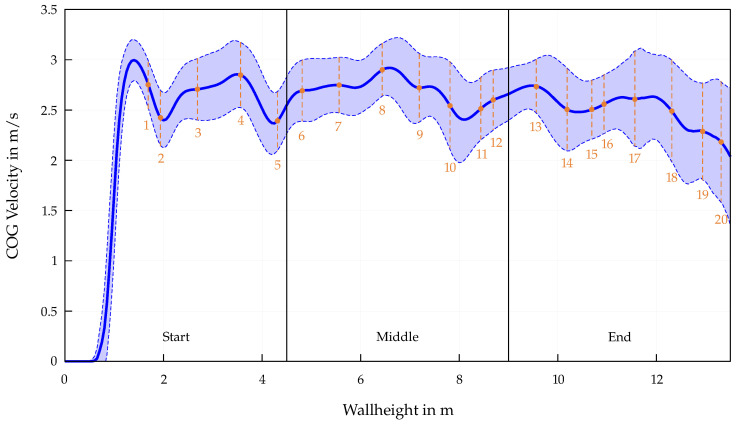
Distribution of the velocity data from the entire dataset, plotted as a function of wall height according to mean (straight line) and standard deviation (dashed line and filled area). The orange dots indicate the positions of the 20 hand holds. In addition, the separation of the Speed Climbing wall into start, middle, and end sections is marked.

The end section of the Speed Climbing wall is also characterized by jumps and skipping holds, which lead to drops in speed, as well as a consequent increase in split times, throughout this entire segment. To illustrate the relationship with the athletes’ split times, the distribution of the velocity data from the entire dataset is presented in Figure 3. For each 5 cm interval from the bottom to the last hold of the wall, the velocities of the athletes in the mentioned dataset are sampled, and the mean and standard deviation are calculated at each point. Additionally, the positions of the 20 hand holds are marked with orange dots, linking the calculated split times with characteristic key transition points in the velocity profile.

**Figure 4:**
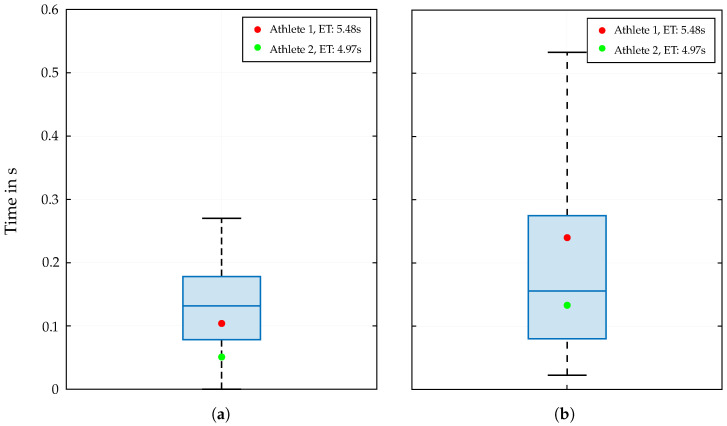
Distribution of the reaction time $t_{r}$ (**a**) and jump time $t_{j}$ (**b**) for the analyzed dataset. The red and green dots indicate the corresponding values of two athletes with different morphological characteristics and performance outcomes.

The results of the reaction time $t_{r}$, measured from the manually determined start signal to the actual movement of the athlete and its COG, show a remarkable high maximum value of 0.27 s (Figure 4a). For the jump time $t_{j}$, the data points correlate with the number of executed steps ahead of the final jump to the buzzer, where an additional intermediate step leads to time losses.

It should be noted that for all the results shown and described, the limited frame rate of the videos (25–30 Hz) must be taken into account, as this particularly affects the accuracy of the split time measurements.

### 3.2. Limb Coordination Analysis

**Figure 5:**
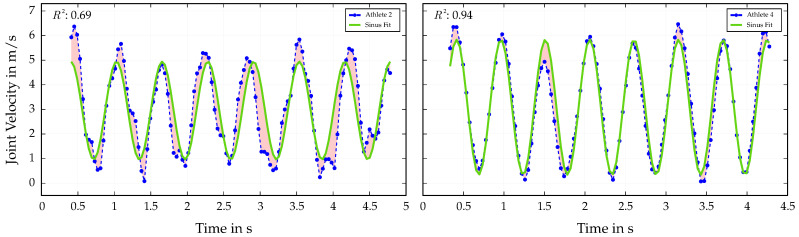
Comparison of fitted sinusoidal models for the right foot velocity data of two athletes (see Table 2, Athletes 2 and 4). Each figure shows the original velocity data (blue), the fitted sinusoidal model (green), and their residuals (red).

As already mentioned, the velocity trajectories of hands and feet are analyzed to investigate the athletes’ limb coordination during Speed Climbing movements. The fitted sinusoidal model (Equation (8)) is used to evaluate the velocity profiles of hands and feet, providing insights into the athletes’ movement patterns and coordination. The results of this analysis are presented in Figure 5, showing the fitted sinusoidal models for the right foot velocity of two athletes with different performance outcomes.

It can be seen that a harmonious movement can be identified for both athletes and that the fitted sinusoidal model describes the velocity data more or less well. However, considering the coefficient of determination $R^{2}$ and the residuals between data points and fit, differences in the execution of the movements can be observed. The athlete with the better performance (Table 2, Athlete 4) shows a more consistent and stable velocity profile, with a higher $R^{2}$ value (0.94) compared to the other athlete ($R^{2}$ = 0.69) (Table 2, Athlete 2). This indicates that the fitted model explains a larger proportion of the variance in the observed data for the better-performing athlete, suggesting a more efficient and coordinated movement pattern.

In the next step, the correlation between the achieved end time and limb coordination is validated to prove the hypothesis that the coordination of individual limb movements correlates with competitive success in Speed Climbing. For this purpose, the following parameters are first determined for the described dataset of 321 runs:

- $R^{2}$: Coefficient of determination of the sinusoidal model;
- Peak distance: Mean of the distances between related peaks of the data and the fitted sinusoidal function;
- Frequency: Dominant frequency of the data calculated using the FFT approach.

In addition to the coefficient $R^{2}$, which reflects the goodness of fit, the averaged peak distance of the data points and the sine model is observed. This parameter intends to provide insights into the coordination of the limbs during climbing movements. A smaller peak distance indicates a more harmonic movement pattern, while a larger peak distance suggests a less-coordinated execution. The use of the mean value is intended to consider the influence of outliers, resulting from potential mistakes in the movement patterns. Finally, the correlation of the performance with the dominant frequency of the velocity data is taken into account.

**Figure 6:**
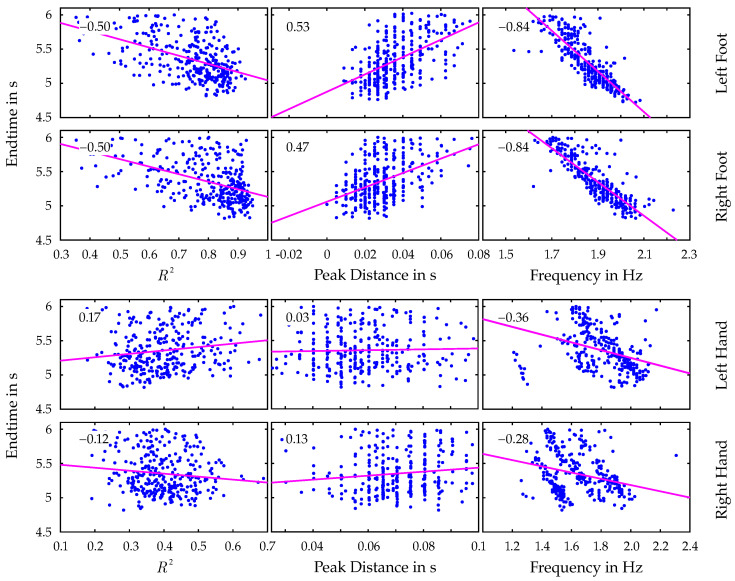
Correlation analysis between limb coordination and end time performance. Each row visualizes the correlation between the achieved end time and the parameters describing limb coordination according to the fitted sinusoidal function.

The correlation between these parameters and the achieved end time is calculated for each limb using Pearson’s correlation coefficient. Parts of the resulting correlation matrices, namely the relations between end time and the mentioned parameters, are visualized in Figure 6.

It is immediately noticeable that there is a striking difference between the correlation results for hands and those for the feet. While significant relations of the limb coordination parameters and the end time can be observed for both left and right feet, the correlation is conspicuously lower for the hands and seems to have no meaningful impact on the athlete’s performance. Another noteworthy observation is the significantly high correlation of the end time with the dominant frequency of the velocity data.

Table 2 shows a detailed comparison of four athletes with different performance outcomes. The *p*-values indicate the statistical significance of the correlation between each athlete’s performance metric and the fitted model. The selection of athletes included one run (1) with a noticeable error (slip), a slow run (2) from an athlete whose personal best times are significantly higher, and runs (3 and 4) from two athletes currently ranked in the World Top 5.

**Figure 7:**
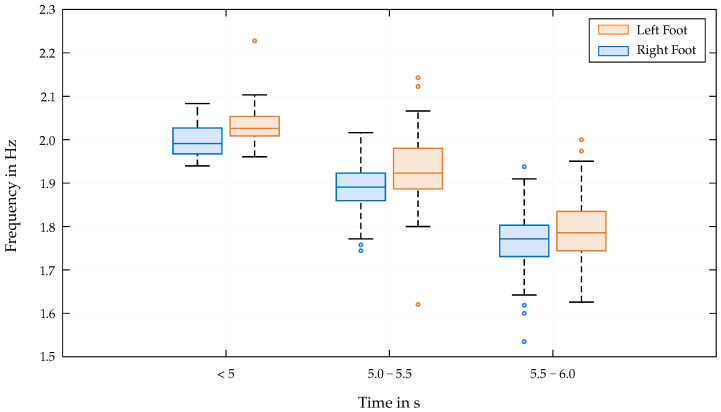
Frequency distribution of the left (blue) and right (orange) ankle velocity data across different end time intervals.

There is a clear trend that better-performing athletes show a higher $R^{2}$ value and a smaller peak distance, indicating a more synchronized movement pattern. The frequency appears to highly influence the performance of the athletes, which is also evident in Figure 6. However, as the end time decreases and a certain level of performance is achieved, the limb frequencies converge to certain ranges and the variability in frequency decreases (see Figure 7).

Finally, the question arises as to whether the limbs not only correlate individually with the end time, but also whether a relationship exists between the coordination of two limbs. Accordingly, the phase shifts of the signals for the velocity data of left and right feet are also considered.

**Figure 8:**
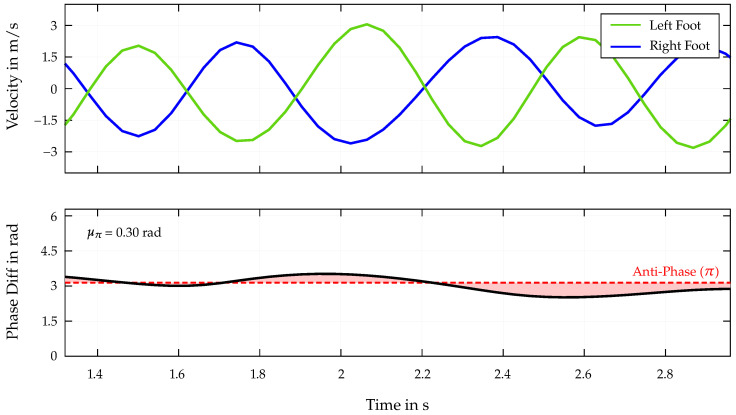
Excerpt (middle section) of the velocity data (*top*) for the left and right foot, as well as the phase differences (**bottom**), with the mean absolute deviation from π ($\mu_{\pi }$).

For this purpose, the raw data of Athlete 4 (Table 2) were preprocessed by subtracting the mean value and filtering the signal using a bandpass filter. The frequency range of the filter was set to 0.5–3 Hz, which covers, including a safety margin, the previously calculated dominant frequencies of the limb velocity for the whole dataset. The instantaneous phase shift distribution φ(t) is then determined as stated in Equations (13) and (14) for left and right foot velocity data. The velocity distributions, as well as the absolute and wrapped phase difference Δφ(t) reduced to the middle section, are shown in Figure 8.

## 4. Discussion

The presented methods and results provide a comprehensive analysis of Speed Climbing athletes’ performance, focusing on a quantitative evaluation of motion patterns in the start, middle, and end sections of the wall by analyzing split times. The runs of various athletes from a dataset filtered by gender, technique, and performance outcome are investigated, revealing significant variations in split times and movement execution. By monitoring the individual split times of all these athletes through normalization, a more accurate assessment of performance analysis is achieved, and the influence of different movement patterns in all sections of the Speed Climbing wall is highlighted. This allows for the generation of a complete overview of the distribution of time intervals and the identification of potential weaknesses or strengths in the athletes’ performance. By evaluating the results of this analysis across the three main sections of the wall, a noticeable increase trend can be observed, whereby the split times in the start section are relatively consistent, with less variability compared to the other sections. This indicates that the start technique, particularly the Tomoa Skip, is well-established among elite athletes and is executed with high efficiency. This special sequence of movements, which was developed about 5 years ago, has become indispensable in Speed Climbing and has thus established itself as the standard technique in the start section [26]. Nevertheless, slight differences can still be recognized when going into more detail. In relation to the physical characteristics of the athletes, the transition time in the area from Hold 3 to Hold 4 (deep squat position) can vary significantly. This result aligns with the findings of Shunko et al. [27], who observed that somatic characteristics significantly affect both the achieved end times by Speed Climbing athletes, as well as the technical strategies they adopt. With targeted training and proper execution [28,29], however, this arrangement of joint angles and the positioning of the COG, originally declared as a disadvantage of this technique, can be optimized to compensate for the resulting drop in velocity.

The transfer from the 5th to the 6th hold and thus the movement into the middle section reveals an interesting distribution of data points, whereby the corresponding boxplot (Figure 2b) shows about 60% of the data concentrated at the median value of $\Delta \hat{t}\approx 0.4\;s/m$, and a significant number of outliers are scattered around this value. Practically, this pattern highlights Hold 6 as a critical transition point. While most athletes manage the move in a similar time, a notable minority experience much slower or faster transitions, which could be due to slight differences in technique or the execution of motion sequences. This insight can help coaches and athletes focus on this phase to reduce variability and improve overall performance.

The closer the athletes are to the end of the route, the greater the distribution of the split times. While the mean values for $\Delta \hat{t}$ for the whole start (0.39 s/m) and middle section (0.38 s/m) are almost identical, the end section shows a significant increase (0.45 s/m). This is mostly attributed to the choice of movement patterns in this section, whereby the athlete has to cover large distances by skipping several holds. By shortening the path length to an almost vertical straight line of the COG, the athlete partially loses speed (see Figure 3). The execution of these movements is reflected in split times, whereby a high degree of variability can be observed (see Figure 2c). A closer look at the course of the velocity in Figure 3 also reveals the increasing deviation from the mean value as the athletes approach the end of the route and thus the influence of different movement sequences on the overall performance. Furthermore, additional overlapping effects may explain the higher split times observed in this final section. In addition to possible signs of fatigue in the last 5 m of the Speed Climbing wall or psychological influences triggered by intense duel with another athlete in the second lane, the specific route design makes section-wise training for movement improvements particularly difficult. Although the route map is adapted by lowering the holds of the end section, the training conditions cannot be compared to those at competition, as it does not account for the transition from the middle section.

In addition to the split times for the respective hand holds, the reaction time and jump time to the end buzzer also play a crucial role in the overall performance, and the results reveal interesting insights. In particular, the distribution of the reaction times over the entire dataset and the location of median (0.13 s) and maximum values (0.27 s) indicate unexpectedly high variability in the athletes’ reaction times. Overall, the range from precise and fast but more risky reactions to significantly slower responses suggests that the athletes’ reaction times are influenced by various factors, including individual training, psychological state, and competition conditions. Similar to the studies of Chen et al. [30] and Hosseini et al. [18], no significant correlation between the reaction time and the performance outcome could be identified.

The last time segment $t_{j}$ measured from passing the last hold to touching the end buzzer also exhibits a high degree of variability. This is mainly attributed to the different movement sequences at the end of the route. A distinction is primarily made between two movement patterns, which are differentiated by the number of steps and the positioning of the feet on the wall starting from Hold 18. While tall athletes prefer to use the previously built-up momentum to jump directly to the buzzer, shorter athletes often need to take an additional step to finish the run.

In addition to this quantitative presentation of the results of the split times analysis, two athletes with different physical characteristics (see Table 1) are separately highlighted. Therefore, their best runs from the mentioned dataset are used to illustrate the influence of individual biomechanics on the performance outcome in Figure 2 and Figure 4. The distribution of these two data points separately for the three wall sections is particularly interesting. The most striking difference in split times can be seen in the start section. Especially when performing the Tomoa Skip, Athlete 2 with the better overall performance (4.97 s) shows a significantly lower split time than Athlete 1 (5.48 s). The poorer efficiency of Athlete 1 executing the Tomoa Skip could be explained by the fact that the athlete used a different technique in the start section (Reza Move) in the years before achieving the mentioned personal best time. This could indicate that the athlete has not yet fully adapted to the new technique, resulting in a less-efficient execution and thus a longer split time. In the middle and end sections, the two athletes show more similar results, with Athlete 2 largely performing at the respective medians of the distributed data points per hold section, while Athlete 1 fluctuates around these values without leaving the interquartile range (IQR).

The individual results for reaction and jump time show a significant correlation with the achieved end time. While physical constitution has no direct influence on the reaction time, parameters such as height and arm span play a crucial role in the choice of the final movements and thus on the jump time.

In general, the results of the split times analysis highlight the importance of individual biomechanics and techniques in Speed Climbing. The evaluation of certain areas of the wall reveals advantages and disadvantages of the movement patterns used, especially for Athlete 1 but also for Athlete 2, which can be used to optimize training methods and thus the performance in future competitions.

The second part of the presented results focuses on the analysis of the limb frequencies of hands and feet in Speed Climbing and their influence on an athlete’s success. As in other sport disciplines, the coordination of limb movements is crucial for achieving optimal performance in Speed Climbing. A sinusoidal model is fitted to the velocity data of hands and feet, allowing for a detailed analysis of the athletes’ movement process. Therefore, the best possible fit of the model to the data with the largest coefficient of determination $R^{2}$ is selected for each signal, and the deviation is first considered objectively by the residuals (see Figure 5). The results show that the fitted model describes the joint velocity data of the athletes’ limbs well, with high $R^{2}$ values indicating a good fit. However, significant differences in the execution of the movements can be observed, especially between athletes with different performance outcomes. The athlete with the better performance shows a more consistent and stable velocity profile, following the estimated mono-frequent sinus more closely. This visual analysis of the deviations of the velocity signal from the fitted model in Figure 5 also highlights possible weaknesses in the athletes’ movement patterns in different section of the Speed Climbing wall, which can be used by trainers and athletes to optimize training methods and improve performance. However, it should also be noted that the distribution of the data points for $R^{2}$ concentrates in a certain direction toward higher values and lower end times. It turns out that for athletes with end times ≤ 6 s, this parameter may no longer be an indicator for their performance. While top athletes have almost perfected common movement patterns in different sections of the wall through intense training, the results of slower athletes show significant deviations from harmonious movements of the limbs. Accordingly, the inclusion of multi-frequent sinusoidal models could be considered in future work to better capture the complexity of the movement patterns in Speed Climbing.

Going into more detail about movement sequences and the dependence on coordination and synchronicity of the limbs, a correlation analysis is performed to investigate the relationship between the athletes’ performance and various parameters. In addition to the already mentioned coefficient of determination $R^{2}$, the mean peak distance of the fitted sinusoidal model as well as the dominant frequency of the velocity data of each limb are considered. Initially, large differences between the correlation results for hands and feet, separately, are observed in Figure 6. While clear and comprehensive correlations can be identified for the feet data, the correlations of all parameters with the hand data are significantly lower. The discrepancy can be attributed to two factors. First, the accuracy of key point detection for hands is generally lower than for feet, especially in the cases of superimposition with the torso. As their covered position cannot be determined precisely in these cases, the location of the keypoints and thus the velocity data are interpolated based on proper points detected on neighboring frames, initially by the human pose detector and subsequently in the postprocessing stage. Secondly, and more importantly, the movement of the hands, unlike that of the feet, lacks a regular pattern. While the feet primarily serve the function of pushing off the wall and stabilizing the body, the hands are repetitively used to pull the athletes’ body toward holds to generate the necessary momentum. The associated increase in contact time with the holds leads to a disharmonious movement pattern. Since the periodicity of the velocity data is not as pronounced as for the feet, the fitting of the sinusoidal model fails, resulting in low $R^{2}$ values and thus a less reliable analysis of the hands’ movement patterns. Accordingly, only the results of the feet data were subsequently taken into account and examined further in more detail.

The correlations of the mentioned parameters with the end time for the lower limbs clearly confirm the second hypothesis of this study, which states that the coordination of individual limb movements correlates with competitive success in Speed Climbing. The results show that better-performing athletes exhibit higher $R^{2}$ values, smaller peak distances, and higher frequencies in their limb movements. The high correlation between end time and $R^{2}$ values indicates that athletes who can maintain a consistent and coordinated movement pattern are more likely to achieve better performance outcomes. The obtained results are consistent with those of Cordier et al. [31], who describe the measured parameters as an entropy index. Figure 5 (right) demonstrates how exceptionally well the data points of a successful athlete adapt to a single-frequency sine wave, resulting in a high $R^{2}$ value close to 1. The smallest deviations from a regular movement due to technical errors in the execution of the movement sequences are also reflected in the rise in peak distances. Despite its correlation with $R^{2}$ (−0.70 for the left foot; −0.77 for the right foot), the consideration of the peak distances is important, as it provides additional insights and helps to identify potential weaknesses in the athletes’ movement patterns. While a significant correlation between lower end times and higher frequencies is not surprising, the distribution of the frequency over the entire time range is interesting. Figure 7 shows that the scatter of frequencies is greater for athletes with poorer performances. This indicates that an increase in frequency could affect the correct execution of the movements, leading to less-efficient patterns or errors. This distribution is substantially lower for the group of athletes achieving remarkable end times below 5 s.

The similarly high correlations for both left and right feet suggest that in addition to the coordination of the individual limbs separately, a synchronized movement pattern of both feet together is also crucial for achieving optimal performance in Speed Climbing. Regarding Figure 8, when looking at the two velocity curves and in the calculated phase differences and $\mu_{\pi }$ values, the athlete moves the two limbs in a almost perfectly coordinated and opposing manner. The mean absolute deviation from $\mu_{\pi }$ is calculated to be 0.3, indicating a high degree of anti-synchronicity between the two limbs during the analyzed movement. When looking at the correlation of these values with the end times for the entire dataset, a relatively low value of 0.24 is obtained compared to the other parameters of the correlation matrix in Figure 6. This indicates that despite existing connections with successful runs, the athletes in the limited dataset for end times < 6 s have generally achieved a high level of coordination and anti-synchronicity between the two feet. Due to the results of the correlation matrix for the upper limbs and the proof of a non-regular movement of the hands in Speed Climbing, an analysis of the anti-synchronized movement of the hands and a synchronous motion of opposite limbs (left hand–right foot; right hand–left foot) is not performed any further.

Finally, it is important to mention the limitations of this study. As already described in the results section, the frame rate of the used recordings is limited to 25–30 Hz. Due to this relatively low temporal resolution, careful consideration is necessary when interpreting absolute split times, as rapid movements may be under-sampled. This limitation affects the accuracy of the presented data, especially for the split times, as these can only be determined with a maximum precision of 1/30 of a second. However, for the methodology and analysis presented in this paper, it is sufficient to provide a first detailed insight into the movement patterns of Speed Climbing athletes. Within a consistent and homogeneous dataset, the presented parameters serve as reliable comparative values to evaluate the athletes’ technique and performance. Accordingly, intensive collaboration with the IFSC is already underway via a Memorandum of Understanding, aiming to obtain direct access to official video materials recorded during competitions. This would improve the reliability of the presented parameters and enable more advanced analyses of the athletes’ performance, such as the precise determination of contact times.

## 5. Conclusions

The presented methods and results provide a comprehensive analysis of Speed Climbing athletes’ performance, focusing on a quantitative evaluation of split times in individual wall sections and a qualitative analysis of limb coordination. The results highlight the importance of individual biomechanics and techniques in Speed Climbing, revealing significant variations in split times and movement execution among athletes. The analysis of the split times, reaction time, and jump time provides valuable insights into the athletes’ performance and can be used to optimize single movement patterns. With the investigation of the velocity data of hands and feet, the study additionally demonstrates the potential of using fitting models to analyze the limb coordination of Speed Climbing athletes. The results reveal that despite the execution of the same movement patterns and techniques, significant differences in the evaluated data can be observed. Therefore, the results are not only relevant for the comparison of athletes with different performance outcomes, but also for the individual evaluation of movement patterns in single sections of the route to uncover potential weaknesses or strengths in the execution. These findings should help coaches and athletes, through video analysis, to focus on specific areas of improvement and optimize training methods accordingly to achieve the best possible outcomes in competitions. Future research plans include the consideration of a female top-athletes dataset and the utilization of higher-quality recordings with increased frame rates to enhance the accuracy of the presented parameters. Furthermore, the integration of multi-frequent sinusoidal models is considered to better capture the complexity of movement patterns in Speed Climbing, as well as the investigation of additional parameters such as contact times to further enhance the understanding of athletes’ performance.

## Figures and Tables

**Table 1:** Listing of certain morphological parameters of the athletes included in the split times analysis in Figure 2.

Athlete	End Time in s	Height in cm	Arm Span in cm	Weight in kg	BMI
1	5.48	168.3	180.0	74.5	26.30
2	4.97	182.0	192.4	74.0	22.34

**Table 2:** Comparison of four athletes with different performance outcomes. The table shows achieved end time, the coefficient of determination $R^{2}$, peak distance, and the dominant frequency of the limb velocity data of left and right feet for each athlete. The *p*-value indicates the statistical significance of the correlation between each athlete’s performance metric and the fitted model (see Figure 6, Right Foot).

Athlete	End Time in s	$R^{2}$		Peak Distance in s		Frequency in Hz	
		Left	Right	Left	Right	Left	Right
1	5.97	0.39	0.48	0.06	0.07	1.63	1.51
2	5.63	0.76	0.69	0.03	0.04	1.7	1.66
3	4.91	0.80	0.91	0.03	0.02	1.66	2.03
4	4.83	0.83	0.94	0.02	0.01	1.69	1.86
***p*** **-Value**	-	≪0.001		≪0.001		≪0.001	

## Data Availability

The dataset was generated solely from the analysis of IFSC recordings, which are publicly accessible on YouTube. However, the data are not available with this paper as we do not have explicit permission to share the recordings.
